# The Larger the Egg, the Safer the Nest: A Study on the Negative Correlation Between Nest Predation Rates and Egg Size of Two Tropical Phasianids in Hainan

**DOI:** 10.1002/ece3.73569

**Published:** 2026-05-10

**Authors:** Yuhan Zhang, Yishuo Ding, Yuxin Xu, Qingling Zeng, Xiaodong Rao

**Affiliations:** ^1^ School of Tropical Agriculture and Forestry Hainan University Danzhou China; ^2^ State Key Laboratory of Animal Biodiversity Conservation and Integrated Pest Management, Institute of Zoology Chinese Academy of Sciences Beijing China; ^3^ Intelligent Forestry Key Laboratory of Haikou City Hainan University Haikou China

**Keywords:** artificial nest, egg size, infrared camera, nest predation, phasianids

## Abstract

Nest predation is the principal cause of breeding failure, with ground nests facing greater predation pressure. Most phasianids are vulnerable to endangerment owing to their relatively large body size, low breeding rate, and tendency to build ground nests. However, research on the nest predation of phasianids largely overlooks egg size, a potential key factor, especially in tropical phasianid nest predation studies. We aimed to investigate the influence of egg size on the predation rate and predator combinations of two phasianids (red junglefowl *Gallus gallus jabouillei* and Chinese francolin *Francolinus pintadeanus* ), which exhibit significant differences in egg size. We installed 167 artificial nests in the sympatric distribution areas of the red junglefowl and Chinese francolin within the Chinese Datian and Bangxi Nature Reserves in Hainan Province between April and September 2023. Eggs of four sizes from artificially reared Japanese quails *Coturnix japonica* , Chinese francolins, domestic chickens, and domestic geese were placed in the nests and monitored with infrared cameras. The results showed that the predation rates for small eggs, medium‐small eggs, medium‐large eggs, and large eggs were 83.3% ± 5.8%, 65.1% ± 7.3%, 51.2% ± 7.8%, and 26.8% ± 6.9%, respectively, exhibiting a decreasing trend, indicating a negative correlation between egg size and nest predation rate. The infrared camera data revealed small‐ to medium‐sized mammals as the primary predators (70.00%: small Indian civet *Viverricula indica* , Asian palm civet *Paradoxurus hermaphroditus* , and wild boar *Sus scrofa* ), followed by birds (20.00%: Greater coucal *Centropus sinensis* and Eastern marsh harrier *Circus spilonotus* ) and snakes (10.00%). Small rodents exerted a weak influence on the nest predation of the two phasianids. Therefore, egg size may contribute to the higher nest predation rate of Chinese francolin than that of red junglefowl. We propose that using experimental eggs matched in size with those of natural eggs improves the accuracy of artificial nest simulation studies. This also provides important empirical evidence to advance our understanding of nest predation mechanisms in terrestrial tropical phasianids, and to inform the development of more effective conservation and management strategies for these species.

## Graphical abstract

There is a lack of research on the predation of tropical pheasant nests. The study conducted artificial nest experiments based on egg size and we have supported the viewpoint that using experimental eggs matched in size with those of natural eggs improves the accuracy of artificial nest simulation studies.

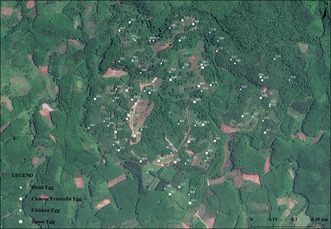

## 1 Introduction

Reproduction is a critical yet energetically costly life‐history stage in birds (Zheng 2012, 2015). Nest predation represents the primary driver of reproductive failure and exerts strong selective pressure on the evolution of avian life‐history traits (Martin 1987, 1992; Lima 2009; Zheng 2012). Phasianids play an important ecological role (Ahmad 2022), yet they typically exhibit large body size, prolonged incubation periods, low reproductive rates, and high sensitivity to environmental perturbations (Wang et al. 2017), which implies a higher risk of endangerment compared to other avian taxa. Given that each reproductive attempt demands substantial parental investment of time and resources, the fitness costs associated with reproductive success or failure are particularly pronounced in phasianids (Wang et al. 2017). Furthermore, most phasianid species construct ground nests (Zheng 2015; Ding et al. 2019), which generally suffer higher predation risk than arboreal or cavity nests (Haskell 2002; Manolis et al. 2002; Minias and Janiszewski 2023), thereby intensifying nest predation pressure during breeding. In light of this high‐investment, high‐risk reproductive dilemma, a thorough investigation into the factors shaping nest predation risk is essential for developing targeted and effective conservation strategies for phasianids.

Existing studies on the influencing factors of nest predation risk in phasianids have primarily focused on nest characteristics such as nest site (Luo et al. 2017; Fu et al. 2020; Li et al. 2023), concealment (Han and Wang 1993; Bu et al. 2019), and structure (Rao et al. 2017), largely neglecting eggs, which are a key factor. Certain egg features, such as egg phenotype (Castilla et al. 2007) and odor (Maier and Degraaf 2001; Coppedge et al. 2007), exert differing influences on the predation rates on bird nests. In addition, the depredated eggs differ in size among nest predator groups (Niehaus et al. 2003; Vazquez et al. 2018). For example, certain small rodents were more likely to prey on small eggs and experienced difficulties in biting through large eggs owing to limitations in their physiological structure (Degraaf and Maier 1996; Rangen et al. 2000; Niehaus et al. 2003). Therefore, egg size may significantly influence the nest predation rate and the predator groups of phasianids.

The high vigilance of phasianids and substantial level of concealment of their nest sites (Rao et al. 2017; Li et al. 2023) limit the direct observation of their nests and eggs under natural conditions, causing the paucity of research on the nest predation of phasianids. Artificial nests provide several advantages, including standardization, repeatability, and flexibility (Major and Kendal 1996; Zanette 2002; Kaisin et al. 2018). They have been effectively applied in studies on the factors affecting nest predation risk (Keyser et al. 1998; Carpio et al. 2014; Holopainen et al. 2024), evaluation of natural nest predation risk, and determination of predator combinations (Mori et al. 2021; Palencia and Barroso 2024). Although several studies have examined the influence of egg size on nest predation risk using artificial nests, the results remain inconsistent. While the majority of studies have reported a negative correlation between egg size and nest predation rate (Janzen 1978; Davison and Bollinger 2000; Coppedge et al. 2007; Oliveira et al. 2013), a few have indicated a positive correlation (Niehaus et al. 2003), and others have found no correlation between the two (Robel et al. 2003; Vazquez et al. 2018). For example, Vazquez et al. (2018) explored the relationship between egg size and nest predation risk of white‐crested elaenia *Elaenia albiceps* by controlling egg size during artificial nest simulation. Results revealed no differences in the predation rates of large and small eggs. Nevertheless, differences in the primary nest predator groups existed among artificial nests containing eggs of different sizes. These studies indicate that using artificial nests containing experimental eggs of unsuitable sizes may not accurately reflect the nest predation rates and predator groups of the target species, and such discrepancies may arise from various factors such as experimental design and predator community composition (Degraaf et al. 1999; Rangen et al. 2000). Ecosystems in tropical regions possess rich biodiversity and complex community structures. Birds in tropical regions face higher nest predation pressure than do those in temperate regions (Robinson et al. 2000; Stutchbury and Morton 2022). The mechanisms underlying the influence of egg size on nest predation risk in tropical phasianids remain unexplored. In‐depth investigation into this aspect aids in elucidating the key factors affecting breeding success in tropical phasianids and offers a novel perspective for understanding the ecological adaptation and anti‐predation strategies of tropical phasianids under high predation stress.

The red junglefowl *Gallus gallus jabouillei* and Chinese francolin *Francolinus pintadeanus* belong to the family Phasianidae of the order Galliformes. Both are ground‐nesting birds, with the eggs of the red junglefowl being significantly larger than those of the Chinese francolin (Zheng 2015; Rao et al. 2023). Although both phasianids have been classified as species of least concern (IUCN 2025), exploring the factors influencing their nest predation may provide an important theoretical basis for the conservation of rare and endangered phasianids in Hainan Province. This study was conducted in two nature reserves in tropical Hainan, China, focusing on the sympatrically distributed red junglefowl and Chinese francolin. Through artificial nest experiments with varying egg sizes combined with infrared camera monitoring, we investigated the influence of egg size on nest predation risk and predator groups in these two phasianid species. We hypothesized the following: (1) Egg size will be negatively correlated with nest predation rate because larger eggs may exclude predation by smaller predators; (2) The primary predator groups will differ among eggs of different sizes. We aimed to expand the understanding of the life‐history theory of tropical birds and provide a theoretical basis and case study for the conservation and management of ground‐dwelling tropical phasianids.

## 2 Materials and Methods

### 2.1 Study Areas and Study Species

The Datian National Nature Reserve, hereinafter referred to as the Datian Reserve, is in Dongfang City in southwest Hainan Province, China (19°05′–17′N, 108°47′–49′E) at an elevation of 30–80 m and covers 1310 ha (Rao et al. 2023). The primary vegetation types within the reserve include seasonal deciduous rainforests, shrublands, low‐elevation tropical grasslands, artificial grasslands, and plantation forests. The Bangxi Provincial Nature Reserve, hereinafter referred to as the Bangxi Reserve, is in Baisha Li Autonomous County in west‐central Hainan (19°22′–24′N, 109°05′–06′E) at an elevation of 100–170 m and covers 361.8 ha (Rao et al. 2023). The primary vegetation types within the reserve include tropical grasslands with sparse trees and shrubs, seasonal deciduous rainforests, and seasonal evergreen rainforests. The Datian and Bangxi Reserves are wildlife nature reserves established primarily for the conservation of Eld's deer *Cervus eldii* . Many terrestrial vertebrates inhabit the two study areas, including Eld's deer, the small Indian civet *Viverricula indica* , greater coucal *Centropus sinensis* , wild boar *Sus scrofa* , and Burmese python *Python bivittatus* (Wang et al. 2002; Fu 2023). Notably, the red junglefowl and Chinese francolin breed sympatrically in both nature reserves (Rao et al. 2023; Zeng et al. 2025).

The red junglefowl is distributed in Guangdong, Hainan, southwestern Guangxi, and Yunnan in China (Rao et al. 2023). The average clutch size is 4.50 ± 1.83 eggs, with white or pale yellow eggshells lacking markings. Egg dimensions are 43.88 ± 1.18 mm × 33.45 ± 0.52 mm (Rao et al. 2023), with an incubation period of 19–21 days (Ding et al. 2019). The Chinese francolin is primarily distributed in Yunnan, Guizhou, Guangxi, and Hainan provinces in China (Zheng 2023). Its average clutch size is 4.10 ± 1.30 eggs, with pale yellow, unspeckled shells measuring 38.00 ± 3.86 mm × 30.29 ± 2.11 mm (Zeng et al. 2025), with an incubation period of approximately 21 days (Ding et al. 2019). From March to September each year, these two phasianid species coexist and breed within the study area, both nesting on the ground in shallow, circular or oval‐shaped depressions.

### 2.2 Artificial Nest Simulation

Artificial ground nest simulation experiments were conducted in the Datian and Bangxi Reserves between April and September 2023. The structures of the natural nests of the Chinese francolin and red junglefowl were simulated in the artificial nests, which had a shallow depression‐like appearance and were constructed using dry leaves, twigs, and feathers as nesting materials. Given the large area of the Datan Reserve, artificial nests were deployed using a kilometer grid system (0.5 km × 0.5 km). Each grid within a group may contain no more than one artificial nest to ensure spatial uniformity and independence among nest locations. For the smaller Bangxi Reserve, the transect method was employed to place artificial nests, with a minimum spacing of 200 m between each nest. In both Datan and Bangxi, suitable habitats for the red junglefowl and Chinese francolin were selected for nest placement. Moreover, eggs of different sizes were randomly distributed across all groups to ensure thorough spatial mixing of treatments for different egg sizes.

The following four groups of artificial nests were established in the two study areas based on the ascending order of the egg size: (1) Small egg group: quail eggs; (2) Medium‐small egg group: Chinese francolin eggs; (3) Medium‐large egg group: domestic chicken eggs (the size is close to that of a natural red junglefowl egg); (4) Large egg group: domestic goose eggs. All experimental eggs used in the present study were unfertilized eggs produced through artificial breeding (All experimental eggs were uniformly sourced from Haikou Luoniushan Agriculture, Industry & Trade Co. Ltd.). A portion of the experimental eggs was randomly selected and weighed using an electronic balance (Senssun EHA901, precision: 0.01 g). The major and minor diameters of the eggs were measured using digital vernier calipers (Guanglu IP67, precision: 0.01 mm) and were used to calculate the egg volume (Hoyt 1979). Egg parameters are summarized in Table 1.

**TABLE 1:** Comparison of parameters in natural egg and experimental egg.

Egg type	Experimental species	*N*	Egg mass (g)	Egg length (mm)	Egg width (mm)	Egg volume (mm^2^)	Sources
Natural egg	Chinese Francolin	40	18.04 ± 4.87	38.00 ± 3.86	30.29 ± 2.11		Zeng et al. (2025)
	Red Junglefowl	9	24.26 ± 0.60	43.88 ± 1.18	33.45 ± 0.52		Rao et al. (2023)
Experimental egg	Japanese Quail	20	10.95 ± 1.02	31.80 ± 1.46	24.92 ± 0.85	10.10 ± 1.02	This study
	Chinese Francolin	20	18.65 ± 1.75	38.84 ± 1.45	29.44 ± 1.02	17.21 ± 1.63	This study
	Chicken	20	59.16 ± 3.07	56.29 ± 1.49	43.77 ± 2.87	55.22 ± 8.08	This study
	Goose	20	155.00 ± 16.22	86.87 ± 6.41	55.98 ± 2.24	138.64 ± 10.04	This study

Two experimental eggs were placed in each artificial nest. The average incubation periods of red junglefowl and Chinese francolin eggs in natural environments are 19 and 21 days, respectively (Zhao 2001; Rao et al. 2023). We set the incubation period for the artificial nest simulation experiments as 21 days and inspected the artificial nests twice during the experiments on Days 10 and 21. Inspection results were classified as follows: (1) Both eggs were intact; (2) One egg was intact, while the other had been depredated (eggshell or traces of predation); (3) One egg was intact, while the other had disappeared (no traces of predation); (4) Both eggs had been depredated; (5) Both eggs had disappeared; (6) Other (e.g., eggs were displaced from the nest). During each inspection, the photographs of the artificial nests were taken and matched to the nest serial numbers. If one or two eggs were present in the nest, the inspection process was continued at the next inspection time point. In the case of predation or the disappearance of both eggs, the experiment was ended after the results had been recorded and the nests had been photographed. Upon the occurrence of damage, displacement, or the disappearance of eggs, new experimental eggs were not introduced, and breeding was considered failed in the nest. Otherwise, the nest was deemed as having achieved breeding success if still found intact during the second visit (Rao et al. 2023).

### 2.3 Infrared Camera Monitoring

A portion of the artificial nests was randomly selected for monitoring with infrared cameras (Yianws L710‐940‐CC) in the entire experiment. Previous studies have indicated that the presence of cameras may influence the behavior of some predators, thereby interfering with nest predation rates (Herranz et al. 2002; Richardson et al. 2009). To control this potential bias, infrared cameras were deployed in this study to ensure uniform distribution across all egg size groups and were randomly installed within each group. This approach maintains the homogeneity of potential camera interference across groups, thereby preventing systematic effects on comparisons of relative predation rates between different treatments. The photograph + video imaging mode was selected, i.e., three consecutive photographs and a 30‐s video were captured. The minimum interval between two triggers (i.e., trigger delay) was set to 0 s to ensure that the next shooting sequence could start without interruption when continuous activity was detected. The camera sensitivity was set to medium (Rao et al. 2023). The cameras were installed on tree trunks at 1.0–1.5 m from the artificial nests and 30–50 cm above ground, with the camera lens facing the nests (Rao et al. 2023). To ensure clear imaging and a wide field of view, the cameras were secured by staking in the absence of conditions for installation, and appropriate camouflaging was performed to reduce disturbances. The serial number, date of installation, date of inspection, and date of recovery of each nest were recorded accurately, and the battery and imaging conditions of each infrared camera were inspected.

### 2.4 Data Analysis

**FIGURE 1:**
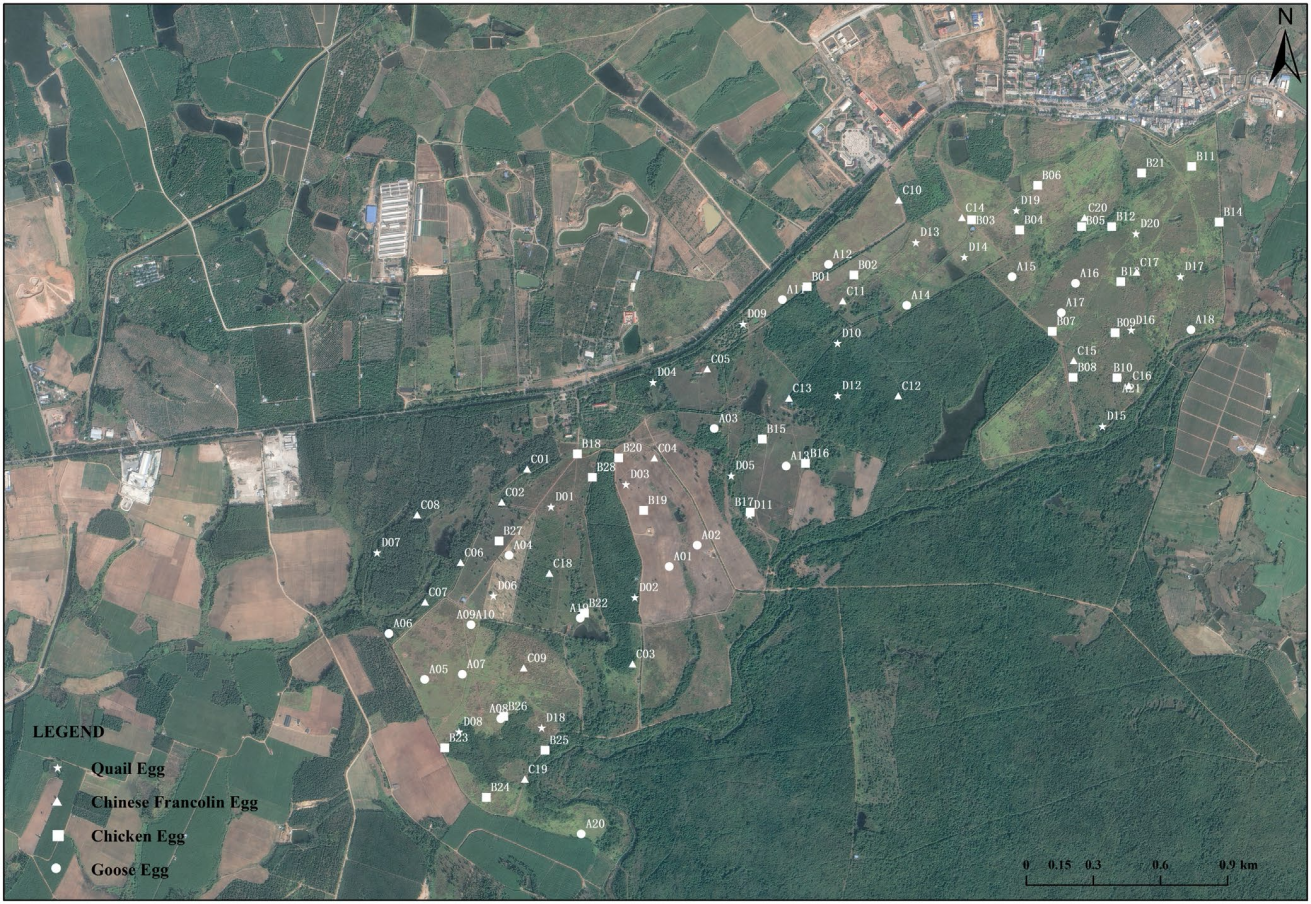
Distribution of artificial nests in Datian National Nature Reserve, Hainan.

**FIGURE 2:**
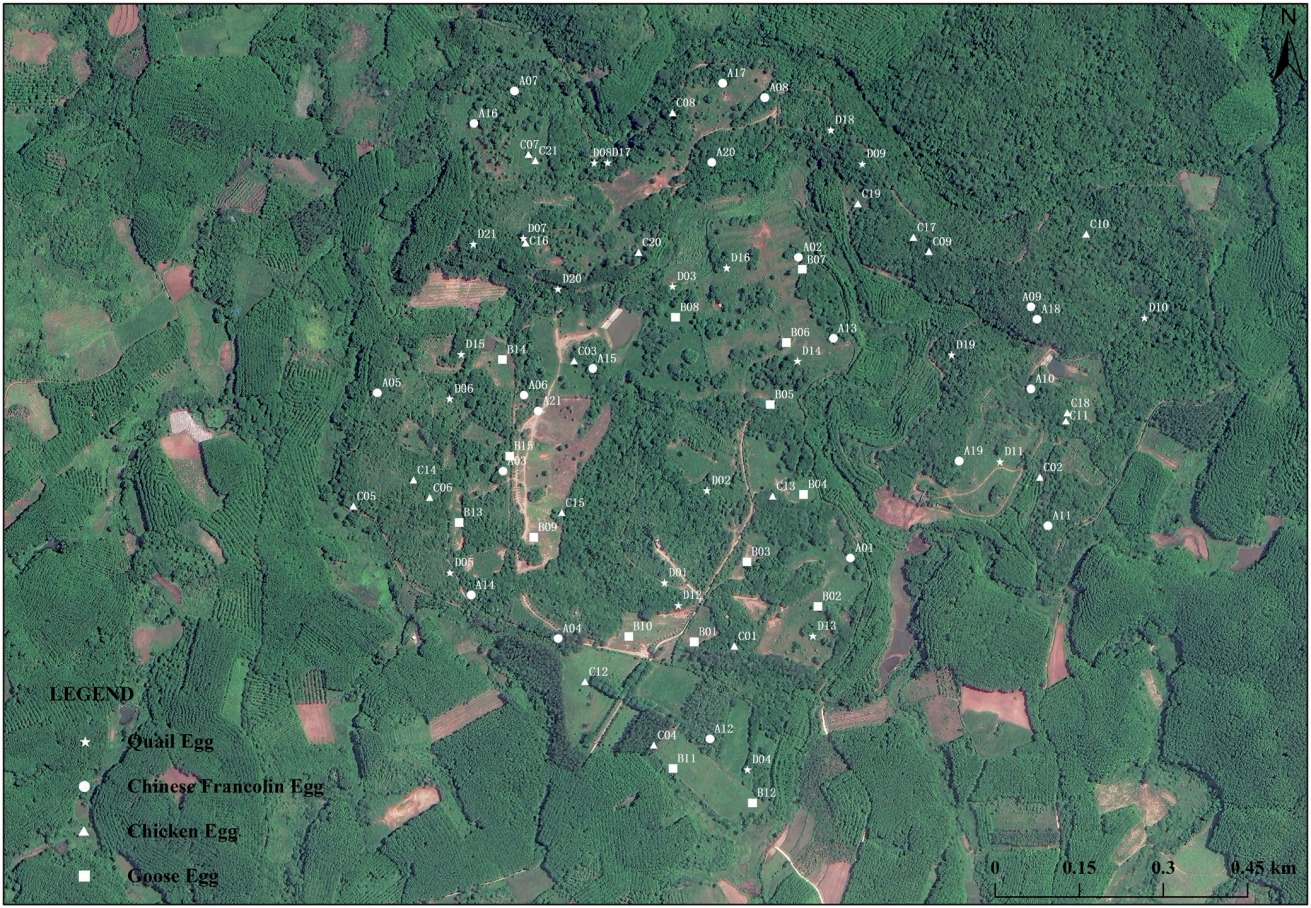
Distribution of artificial nests in Bnagxi Provincial Nature Reserve, Hainan.

Data source: Data were collected from April to August 2023 across two study areas (Area 1: Datian, *n* = 89; Area 2: Bangxi, *n* = 78), encompassing a total of 167 nests (Figures 1 and 2). For each nest, we recorded egg size grade (levels 1–4, representing small, medium‐small, medium‐large, and large, respectively), experimental date, vegetation type (types 1–7, representing low‐elevation tropical grasslands, artificial grasslands, plantation forests, shrublands, seasonal evergreen rainforests, seasonal deciduous rainforests, and tropical grasslands with sparse trees and shrubs, respectively), and experimental outcome (depredated = 1, successful = 0). Sample sizes for the four egg size categories were 42, 43, 41, and 41, respectively.

Variable Processing: Experimental dates are recorded in the “month.day” format (e.g., 3.29 denotes March 29). Prior to modeling, these dates are converted to ordinal days—the ordinal day of the year (e.g., March 29 = Day 88, August 17 = Day 229). The original vegetation type variable consisted of seven levels, some of which had very small sample sizes (e.g., type 4, *n* = 6), and cross‐tabulation with other predictors resulted in empty cells, leading to a rank‐deficient design matrix in standard logistic regression. Based on observed predation rates, we therefore collapsed the seven categories into two groups: a high predation rate group (types 1 and 2, predation rates 73.3%–83.3%) and a low predation rate group (types 3–7, predation rates 33.3%–54.5%).

Statistical modeling: Binary logistic regression was used to examine the effects of various factors on experimental outcomes. The dependent variable was Result (0/1), and the independent variables included egg size grade (categorical variable), study area, and vegetation group. Egg size grade was an ordinal categorical variable, with grade 1 as the reference level. Specifically, the following models were constructed: (1) The main effects model was constructed as: $\text{logit}\left(p\right)=\beta_{0}+\beta_{1}\cdot {\text{Grade}}_{2}+\beta_{2}\cdot {\text{Grade}}_{3}+\beta_{3}\cdot {\text{Grade}}_{4}+\beta_{4}\cdot {\text{Area}}_{2}+\beta_{5}\cdot {\mathrm{Veg}}_{\mathrm{low}}$; (2) To test whether the effect of egg size grade differed between the two study areas, an interaction term between egg size grade and study area was added to the main effects model. The contribution of the interaction term was assessed using the likelihood ratio test (LRT) between the two models; (3) There were differences in the temporal distribution between the experimental date (ordinal date) and certain levels of egg size grade (grade 2 was concentrated between days 69–143, while the other grades were concentrated between days 88 and 229), leading to collinearity when both were included in the regression (GVIF > 6). To avoid interference between coefficients, the ordinal date was not included in the main effects model. The effect of date on the experimental outcomes was reported using a separate logistic regression model; (4) Collinearity was diagnosed using the generalized variance inflation factor (GVIF), goodness‐of‐fit was assessed using the Hosmer‐Lemeshow test, and discriminative ability was evaluated using the area under the receiver operating characteristic curve (AUC). Pairwise comparisons between grades were performed using the emmeans package with the Tukey method for multiple comparison correction. All analyses were conducted in R version 4.5.3, utilizing the following packages: car, emmeans, logistf, generalhoslem, and pROC.

Infrared camera data processing: The infrared camera data were processed to screen records of imaging triggered by animals, from which records of nest visits by animals were selected to identify the nest predators. Animals that attempted to come into contact with the eggs and those that moved or depredated the eggs were regarded as having engaged in predation events. Behaviors involving the damage of egg integrity and removal of eggs were regarded as successful predation, and animal species that engaged in these behaviors were defined as nest predators (Noske et al. 2008). The predator species were identified with reference to the Chinese Wildlife Manual of Mammals (Smith and Xie 2009), The CNG Field Guide to the Birds of China (Liu and Chen 2021), and A Checklist on the Classification and Distribution of the Birds of China (Fourth Edition) (Zheng 2023). Different predators produce distinct traces after preying on bird eggs: snakes typically leave no remains; mammals generate small, scattered eggshell fragments; in contrast, avian predators produce larger fragments and often create conspicuous holes in relatively intact eggshells. For predation events that were not captured on the cameras, predator species were inferred based on the traces of predation (Klug et al. 2010).

## 3 Results

### 3.1 Artificial Nest Predation Rates

**FIGURE 3:**
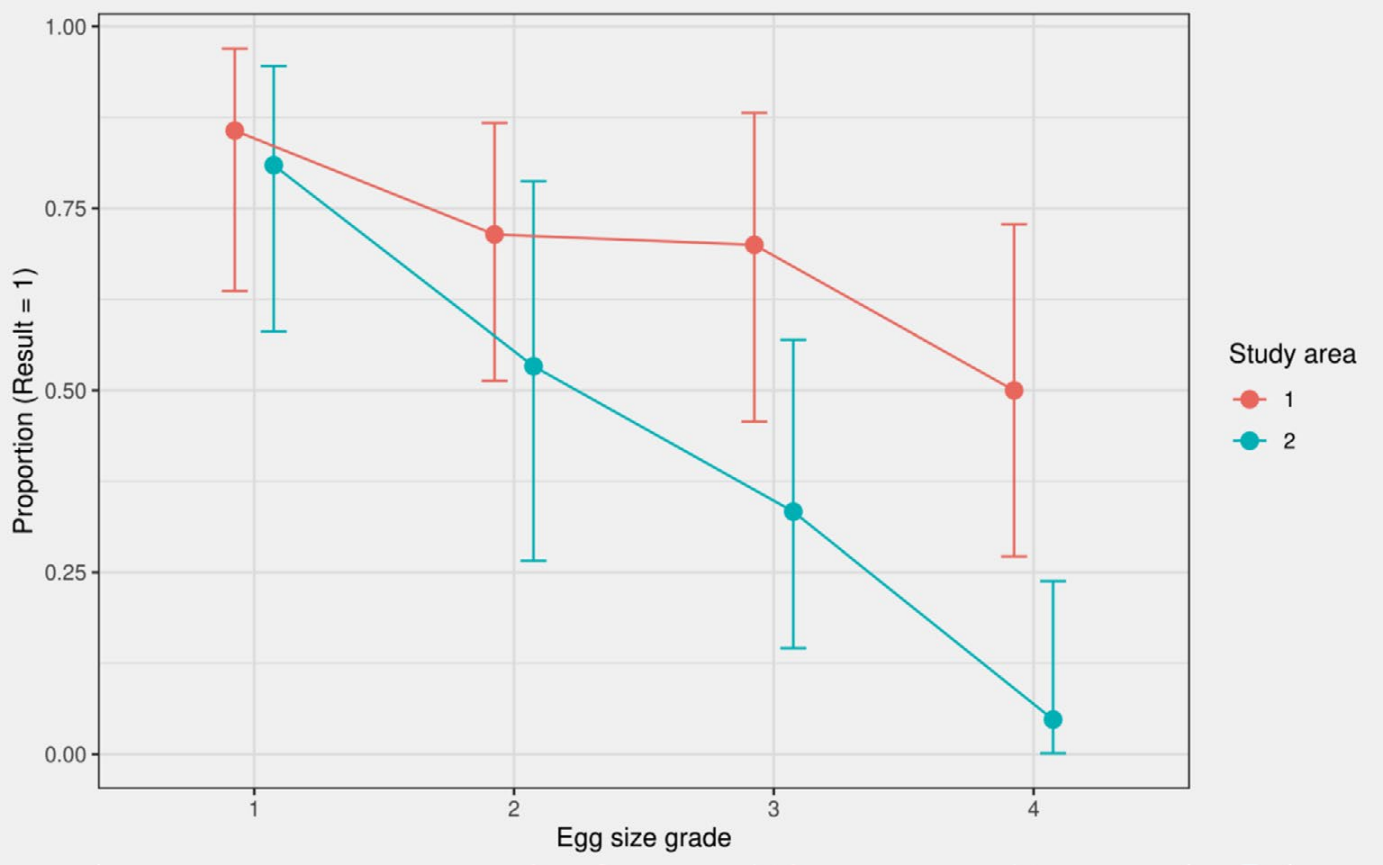
Predation rates by egg size grade and study area (Error bars show 95% exact binomial confidence intervals).

The predation rates (± SE) for the four egg size grades (grades 1–4) were 83.3% ± 5.8%, 65.1% ± 7.3%, 51.2% ± 7.8%, and 26.8% ± 6.9%, respectively, showing a clear decreasing trend. The predation rates in both areas decreased with increasing grade. In Area 1, the rate decreased from 85.7% at grade 1 to 50.0% at grade 4, a decline of 35.7%; in Area 2, the rate decreased from 81.0% to 4.8%, a decline of 76.2%, with a much faster rate of decline than that in Area 1 (Figure 3). Detailed predation rates (±SE) and sample sizes for each combination of study area and egg size grade are provided in Table 2.

**TABLE 2:** Predation rates (±SE) and sample sizes for each combination of study area and egg size grade.

Study area	Grade1 (small egg)	Grade2 (medium‐small egg)	Grade3 (medium‐large egg)	Grade4 (large egg)
Area 1 (Datian)	85.7% ± 7.6% (*n* = 21)	71.4% ± 8.5% (*n* = 28)	70.0% ± 10.2% (*n* = 20)	50.0% ± 11.2% (*n* = 20)
Area 2 (Bangxi)	81.0% ± 8.6% (*n* = 21)	53.3% ± 12.9% (*n* = 15)	33.3% ± 10.3% (*n* = 21)	4.8% ± 4.7% (*n* = 21)

After segmenting the ordinal day by quartiles, the predation rate (±SE) gradually decreased from 69.7% ± 4.9% on days 69–109 (*n* = 89) to 23.3% ± 7.7% on days 190–229 (*n* = 30), indicating that the later the experimental date, the lower the predation rate. In the combined vegetation groups, the high predation rate group was 75.0% ± 5.1% (*n* = 72), and the low predation rate group was 43.2% ± 5.1% (*n* = 95).

GVIF values in Model 1 were 1.11 for egg size grade, 2.72 for study area, and 2.72 for vegetation group, indicating that collinearity was within an acceptable range. The Hosmer‐Lemeshow test did not reject the goodness‐of‐fit assumption (*χ* ^2^ = 5.83, df = 3, *p* = 0.120). The area under the ROC curve (AUC) was 0.788, and the optimal cutoff threshold of 0.443 yielded a sensitivity of 82.1% and a specificity of 63.9%.

**FIGURE 4:**
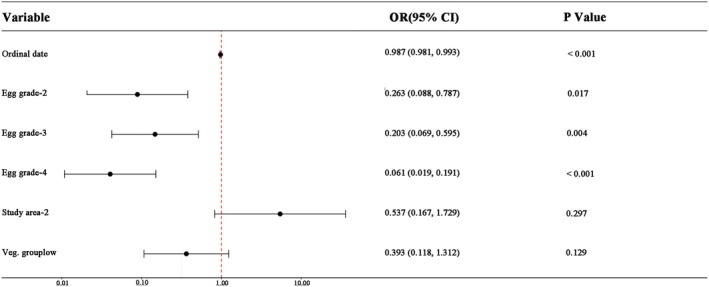
Forest plot of odds ratios for variables in Model 1 (dashed line indicates OR = 1; reference categories: Egg size grade‐1, Study area‐1, Veg. group high).

The odds ratios (ORs) for the three grades were all far below 1, with confidence intervals not crossing 1, and the OR values monotonically decreased from grade 2 to grade 4 (Figure 4). With grade 1 as the reference, the predation odds for grade 2 decreased to 26.3% of that of grade 1 (*p* = 0.017), for grade 3%–20.3% (*p* = 0.004), and for grade 4%–6.1% (*p* < 0.001). After controlling for grade, neither study area nor vegetation group reached a statistically significant level.

In the single‐factor logistic regression of date, for each one‐day delay in ordinal day, the predation odds decreased by approximately 1.3% (OR = 0.987, 95% CI: 0.981–0.993, *p* < 0.001). The predicted predation probability decreased from 74.6% on day 69%–26.3% on day 229.

Further pairwise comparisons using the Tukey correction showed that the differences between grade 1 and grade 3 (OR = 4.92, *p* = 0.019), grade 1 and grade 4 (OR = 16.42, *p* < 0.001), and grade 2 and grade 4 (OR = 4.31, *p* = 0.021) reached statistical significance. The differences between grade 1 and grade 2 (OR = 3.81, *p* = 0.080) and between grade 3 and grade 4 (OR = 3.34, *p* = 0.078) were marginally significant. No significant difference was found between grade 2 and grade 3 (OR = 1.29, *p* = 0.952). Overall, grade 1 was clearly distinguished from grades 3 and 4, and grade 2 was clearly distinguished from grade 4, whereas the differences between adjacent grades (grade 1 vs. grade 2, grade 2 vs. grade 3, and grade 3 vs. grade 4) were relatively small. This pattern is consistent with the characteristics of an ordinal categorical variable—the effect differences between adjacent levels are limited, but the cumulative effect across nonadjacent levels is sufficient to reach statistical significance.

LRT showed that the interaction term between egg size grade and study area did not reach statistical significance (*χ* ^2^ = 6.26, df = 3, *p* = 0.100), but was close to marginally significant. Among the interaction terms, the coefficient for the grade 4 by Area 2 interaction was the largest ($\hat{\beta }=-2.83$, p=0.044), indicating that the effect of grade 4 was much stronger in Area 2 than in Area 1. In the pairwise comparisons stratified by study area, no significant differences were found among grades within Area 1, which is consistent with its relatively gentle decreasing trend. In Area 2, the differences between grade 1 and grade 3 (OR = 8.50, *p* = 0.016), grade 1 and grade 4 (OR = 85.00, *p* < 0.001), and grade 2 and grade 4 (OR = 22.86, *p* = 0.033) reached statistical significance.

### 3.2 Nest Predators and Potential Nest Predators

**FIGURE 5:**
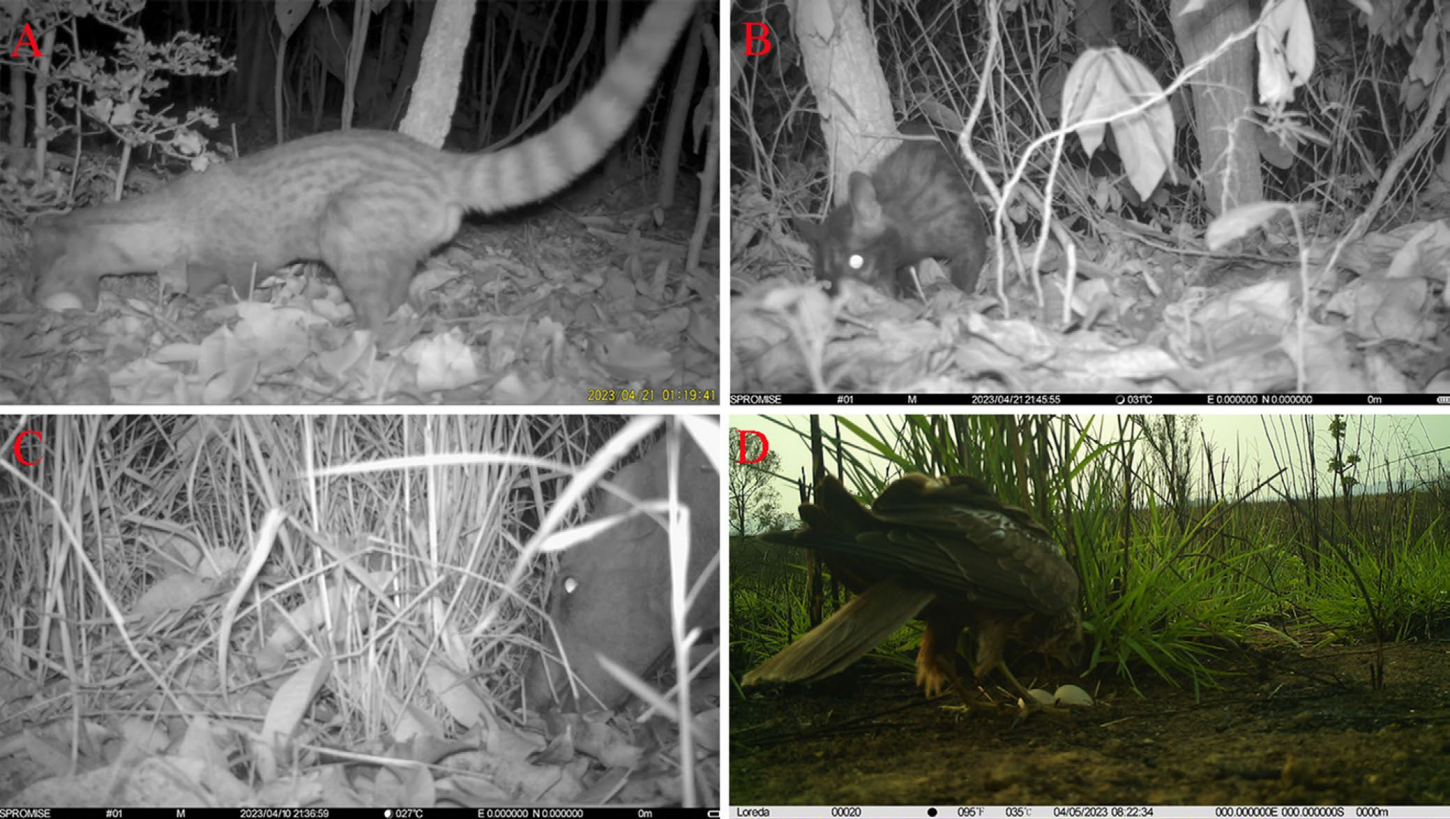
Predators are feeding on eggs in artificial nests (A: Small Indian Civet; B: Northern Palm Civet; C: Wild Boars; D: Eastern Marsh Harrier).

We monitored 36 and 38 artificial nests in the Datian and Bangxi Reserves, respectively, using infrared cameras. Ten cases of nest predation were recorded, two of which were secondary (i.e., the same nest was preyed upon again after the initial predation event, targeting any remaining nest contents). Mammals constituted seven predation cases (70.00%), birds accounted for two (20.00%), and reptiles were involved in one (10.00%). The monitoring in the Datian Reserve revealed that medium‐small egg group had one case of predation by the greater coucal. In medium‐large egg group, there was one case of predation by the Eastern marsh harrier *Circus spilonotus* (Figure 5D), one by the small Indian civet (Figure 5A), followed by secondary predation by the Pallas's squirrel *Callosciurus erythraeus* , one by the Asian palm civet *Paradoxurus hermaphroditus* (Figure 5B), and one by the wild boar (Figure 5C). In large egg group, there was one predation case by the small Indian civet and one by the Asian palm civet, followed by secondary predation by the Pallas's squirrel. No predators were recorded in small egg group. In the Bangxi Reserve, only one case of snake predation was recorded in medium‐large egg group, while no predators were recorded in small, medium‐small, or large group. In the Datian Reserve, camera recordings showed that, in two nests, the residual eggshell fragments were depredated by the Pallas's squirrel after the nest had been depredated by the small Indian civet. For nests with no recorded predators, traces of predation (eggshell fragments or traces of animal digging) were not observed, leading to the speculation that the eggs had been swallowed whole by a snake (Barends and Maritz 2022).

**FIGURE 6:**
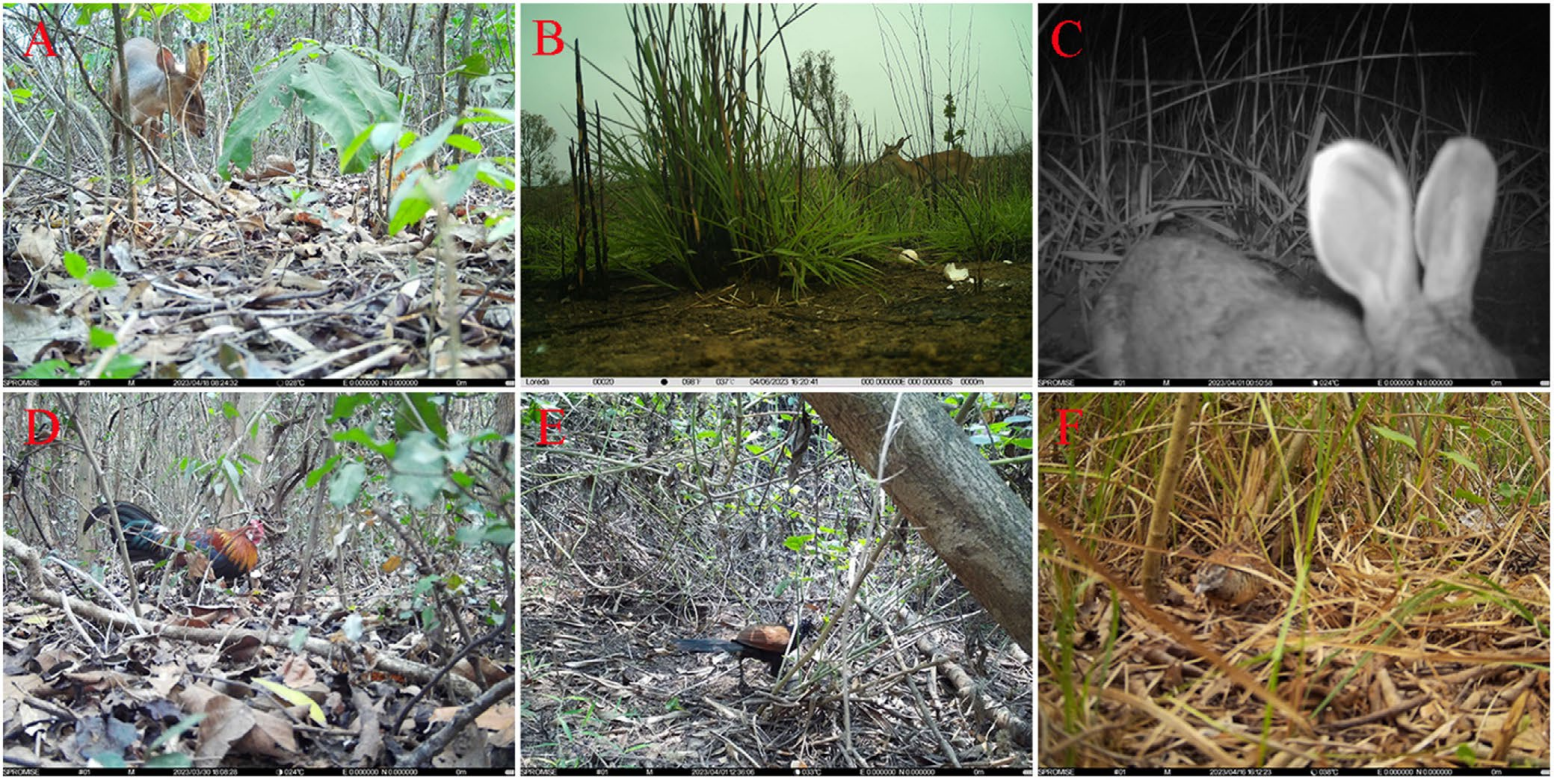
Some visitors near artificial nests (A: Norther Red Muntjac; B: Hainan Eld's deer; C: Hainan Hare; D: Red Junglefowl; E: Greater Coucal; F: Barred Buttonquail).

Furthermore, the infrared cameras recorded the activities of multiple wild animals, including mammals and birds, near the artificial nests. A total of 114 animals were recorded in the Datian Reserve, including six mammals, several rodents that could not be identified, and 12 bird species. In the Bangxi Reserve, 58 animals were recorded, which included five birds and four mammals. Among these nest visitors, the wild boar, small Indian civet, and greater coucal are species with documented predation records. Animals of the family Sciuridae (including the maritime striped squirrel *Tamiops maritimus* , pallas's squirrel, and red‐hipped squirrel *Dremomys pyrrhomerus* ) may be potential nest predators, though their ability to prey on the relatively large eggs of phasianids may be limited. Most of the remaining visitor species are herbivorous or feed on small invertebrates and do not exhibit egg‐predation behavior. Images of some visitors are shown in Figure 6.

## 4 Discussion

This study found that experimental egg size was negatively correlated with nest predation rate, and that predation rate tended to decrease with later experimental dates. Different combinations were observed in the nest predators of the experimental eggs. Medium‐sized mammals were the primary nest predators, followed by birds, while snakes may be significant predators.

Our results indicated that small eggs faced a higher predation risk than did big eggs within the same spatial scale. This is consistent with the results of most other studies and further validates the negative correlation between egg size and nest predation rate (Janzen 1978; Davison and Bollinger 2000; Coppedge et al. 2007; Oliveira et al. 2013). For most predators, small‐sized and thin‐shell eggs are easier to prey on than those with large sizes and thick shells (Picman et al. 1993; Major and Kendal 1996; Bayne et al. 1997). Therefore, small eggs have a greater tendency to be depredated by a wider variety of predators than big eggs. Small mammals are the primary predator group for ground nests (Maxson and Oring 1978; Söderström et al. 1998; Degraaf et al. 1999). Certain small predators prey on large eggs through special behaviors. For example, the Eastern chipmunk *Tamias striatus* utilizes the lever effect to destroy large eggs, implying a predation event (Craig 1998). However, most small rodents cannot bite through large eggs and spend significant time on predation owing to limitations in their mouth structure and size (Degraaf and Maier 1996; Rangen et al. 2000; Niehaus et al. 2003).

However, this study differs from some reports that found no significant association or even a positive correlation between egg size and predation rate (Robel et al. 2003; Vazquez et al. 2018; Bravo et al. 2020). This discrepancy likely stems from differences in predator community composition across study regions and their specific predation preferences for egg size. Vazquez et al. (2018) reported that the primary predators of small eggs were small mammals, while birds primarily preyed on large eggs. Similarly, the composition, abundance, and distribution of predator communities differ across study areas owing to varying habitat types (Vazquez and Amico 2023; Angoh et al. 2024; Ferrante et al. 2024). Therefore, the heterogeneity in research findings essentially reflects the interactions among habitat type, predator community, and egg size‐dependent predation rates. Taking the study by Bravo et al. (2020) in agricultural landscapes as an example, the primary predators were corvids. These birds are capable of preying on eggs regardless of size and occur at high abundances in such habitats. However, larger eggs may have attracted more predation events due to their greater conspicuousness, leading to the observed positive correlation between egg size and predation rate in their study. In contrast, the present study encompasses multiple habitat types and features a more complex predator community composition. Within the local predator assemblage, it is likely dominated by taxa that prefer smaller eggs, such as certain small mammals. These findings indicate that the structural specificity of predator communities in habitats with varying levels of biodiversity is a key factor determining how egg size influences nest predation risk.

Artificial nests are common in research on the predation of bird nests (Keyser et al. 1998; Krüger et al. 2018; Faria et al. 2022). However, certain differences between artificial and natural nests may bias results (Berry and Lill 2003; Burke et al. 2004; Thompson and Burhans 2004). For example, the activities of parent birds are regarded as a crucial influencing factor of predation risk. Therefore, the predation rates of artificial nests may be low owing to the absence of parent birds (Bayne et al. 1997; Robel et al. 2003). In addition, the varying odors of nesting materials and eggs may cause inaccuracies in the recorded predator groups, which rely primarily on olfactory cues (Rangen et al. 2000; Maier and Degraaf 2001; Coppedge et al. 2007). In this study, we minimized odor variation among experimental eggs by standardizing their procurement and handling. However, artificial nests still have limitations in simulating the odors associated with parental behavior and natural nesting materials. Certain studies have demonstrated that using inappropriately sized experimental eggs skews the results (Degraaf et al. 1999; Coppedge et al. 2007; Oliveira et al. 2013). This viewpoint has been further validated by the present study. Notably, the predation rates of natural red junglefowl and Chinese francolin nests in the same area were 31.25% (Rao et al. 2023) and 72.73% (Zeng et al. 2025), respectively, which did not differ significantly from those in the domestic chicken (51.22%) and Chinese francolin (65.12%) egg groups among the artificial nests of the present study. Our results suggest that the degree of similarity in sizes between experimental and natural eggs directly determined the magnitude of differences between predation rates. This implies that a closer match in egg size led to small differences in the predation rates between artificial and natural nests. The color of eggs and their camouflage effect are also important influencing factors. For instance, in this study, quail eggs would theoretically be expected to provide better background matching compared to the pure white eggs in other groups. However, the highest predation rate was recorded for quail eggs in our study. This finding, on the contrary, provides strong support for our core conclusion that predation differences are closely associated with egg size. This result indicates that, within the study system, the impact of egg size on predation risk may outweigh the protective effects provided by color camouflage.

Our infrared camera data revealed that medium‐sized mammals were the primary nest predators, followed by birds and snakes. This finding is similar to the results of previous studies involving the nest predator groups of certain phasianids (Melville et al. 2014; Cheng et al. 2018; Lin et al. 2023; Palencia and Barroso 2024). Rao et al. (2023) used an artificial red junglefowl nest simulation experiment within the same study areas and revealed that rodents were among the key predators. The present study indicated that the primary nest predators were medium‐sized mammals that could easily prey on domestic chicken eggs, such as the wild boar, small Indian civet, and Asian palm civet. Bird predators recorded by the infrared cameras included the greater coucal and Eastern marsh harrier, which preyed on Chinese francolin and domestic chicken eggs, respectively. Birds are an important predator group of ground nests (Angelstam 1986; Krüger et al. 2018; Bravo et al. 2020). For medium‐sized or large omnivorous or carnivorous bird predators, egg size does not pose limitations to predation owing to the ability of birds to pierce eggs with their sharp beaks. For instance, crows may carry small eggs away with their beaks and pierce large eggs that cannot be carried and consume the egg contents (Montevecchi 1976).

In early research on nest predation, small passerines were common study species, and quail eggs were frequently used as the experimental eggs in artificial nests (Danielson et al. 1997; Marini and Melo 1998). However, quail eggs are bigger and have a thicker shell than the eggs of most passerines (Degraaf and Maier 1996). Degraaf et al. (1999) pioneered the experimental use of house sparrow *Passer domesticus* eggs, stating that using quail eggs as experimental eggs in artificial nests may cause the overlook of many small rodent predators. In subsequent research on the influence of egg size on nest predation risk, quail eggs have been used as large eggs, with passerine eggs serving as small eggs (Niehaus et al. 2003; Robel et al. 2003; Coppedge et al. 2007). Nevertheless, compared with those of passerines, galliforms usually produce large eggs (Zhao 2001; Zheng 2012). The present study differed from previous research in that quail eggs were used as the smallest (in size) experimental eggs. Although the infrared camera data indicated that multiple nests had been visited by small rodents, predation events by small rodents were not recorded at all experimental nests, including the quail egg groups. In addition, our imaging data showed that multiple nests were visited by Pallas's squirrels; however, there were no records of direct predation of eggs by these animals. Only one nest of each of the domestic chicken and goose egg groups experienced the secondary predation of the residual eggshells by Pallas's squirrels following predation by the small Indian and Asian palm civets, respectively. These results suggest that the influence of small rodents on the predation rates of the red junglefowl and Chinese francolin nests may be smaller than that of medium‐sized mammals, birds, and snakes.

In addition to the aforementioned predators, many other wild animal species visited the artificial nests; however, these animals did not prey on the experimental eggs. The nest visitors primarily fed on invertebrates or plants. Predation events that were not captured by the infrared cameras had no traces of predation, suggesting that snakes were the primary nest predators in these events. This is because the effect of egg size on the snake predation of eggs is limited. Moreover, snakes, in contrast to birds and mammals that usually leave eggshell fragments after predation, do not leave traces of predation (Marini and Melo 1998; Klug et al. 2010). However, these results may not be accurate. First, the predators may remove the eggs from the nests (Larivière 1999; Rao et al. 2023). For example, crows carry small eggs away with their beaks. In such situations, even non‐snake predators would not leave traces of predation (Montevecchi 1976). Second, traces of predation vary. Changes may occur in these traces over time or owing to rainfall, microbial decomposition, and animal movement. Furthermore, certain nests may experience secondary predation (Stanton 1944), which might also cause changes in predation traces. For instance, two cases of secondary predation of residual eggshells by the Pallas's squirrel were recorded in the present study. Therefore, for nests where predators were not captured on imaging data, certain biases may be introduced when the type of nest predator is inferred solely based on traces of predation.

In addition to egg size, this study also found that seasonal variation exerted a relatively significant influence on predation rates, specifically manifested as a gradual decline in predation rates with the progression of dates. This trend may primarily be attributed to the following mechanisms. First, as the breeding season progresses, the number of natural nests gradually increases and accumulates, leading to a rise in overall nest density within the environment. Under relatively stable predator populations, the greater the number of concurrent nests, the more the predation risk for individual nests is effectively “diluted”, ultimately resulting in an overall decline in predation rates (Schmidt and Whelan 1999; Lima 2009). Second, the activity patterns, foraging intensity, or population size of some predators may themselves vary with the season, which in turn can affect nest predation risk (Thompson 2007). Additionally, as the season progresses, increased vegetation cover enhances nest concealment, which may also reduce the likelihood of artificial nests being detected by predators (Wilson et al. 2005; Dagan and Izhaki 2020). However, the influence of date on predation rates was global, meaning that all nests experienced a similar directional change in seasonal risk. This indicates that even within the same period, nests containing smaller eggs still faced a higher predation risk than those with larger eggs. Therefore, seasonal variation did not mask the dominant effect of egg size on predation risk, nor did it weaken the core conclusion of this study that egg size was the primary factor influencing predation rates.

Additionally, although the difference in predation rates between the two study sites was not statistically significant in this study, substantial variation was observed. Pairwise comparisons revealed that the magnitude of the difference in predation rates between sites increased with egg size, thereby accounting for the considerable overall variation between regions. Nest predation rate is positively correlated with the size of the breeding area (Maag et al. 2022). Differences in predator community composition at different spatial scales constitute a key contributing factor to differences in nest predation rates (Vazquez and Amico 2023; Angoh et al. 2024). The Datian Reserve has a larger forest area and higher species richness than does the Bangxi Reserve. Therefore, the species number and population sizes of mammalian and bird species capable of preying on large eggs are larger in the Datian Reserve than in the Bangxi Reserve. On the other hand, experimental dates in the Bangxi Reserve were generally later than those in the Datian Reserve. As noted earlier, the progression of the season may lead to a gradual decline in predation rates through mechanisms such as predator satiation via increased nest density and greater vegetative concealment. This temporal offset may have further reduced nest predation rates in the Bangxi Reserve under seasonal effects, thereby, to some extent, amplifying the differences in predation rates between the two sites within the larger egg size categories. This represents one of the main limitations of the present study. Future research should aim to conduct cross‐regional comparisons within the same time window to more precisely disentangle spatial variation in predation rates and its associations with ecological and environmental factors.

In the present study, we systematically evaluated the influence of egg size on the nest predation rates and predator groups of two tropical phasianids by controlling experimental egg size in artificial nests. Our results indicated that nests with small eggs faced a greater risk of nest predation than did those containing large eggs. This phenomenon suggests that egg size is a key factor contributing to the higher nest predation rate of the Chinese francolin than the red junglefowl. Medium‐sized mammals were the primary nest predators of both phasianids, followed by medium‐sized birds and snakes. These predator groups handled relatively large eggs. Nevertheless, small mammals still encounter substantial difficulties when preying on the two phasianid species' eggs due to their respective egg sizes. Consequently, the impact of this predator group on the nest predation rates of the two phasianid species is limited. The present study has a limitation in that the number of clearly recorded predation events was limited. Therefore, the expansion of the scale and density of infrared camera deployment is essential in subsequent research to enable the detailed analysis of the predator groups corresponding to different egg sizes, as well as to facilitate an in‐depth investigation into other potential factors affecting nest predation risk and predator combinations of phasianids.

## Author Contributions

**Yuhan Zhang:** writing – original draft (equal). **Yishuo Ding:** formal analysis (equal), resources (equal). **Yuxin Xu:** data curation (equal), resources (equal). **Qingling Zeng:** data curation (equal), resources (equal). **Xiaodong Rao:** formal analysis (equal), funding acquisition (lead), project administration (lead), resources (lead), writing – review and editing (lead).

## Funding

This work was funded by the National Natural Science Foundation of China (No. 32360250 to X.R.), the Hainan Institute of National Park and Hainan Province Science and Technology Special Fund (HINP, KY‐24ZK02, ZDYF2023RDYL01 to X.R.), and the Hainan Province Graduate Innovative Research Project (No. Qhys2024‐157 to Q.Z.).

## Conflicts of Interest

The authors declare no conflicts of interest.

## Supporting information


**Table S1:** Parameters of the experimental eggs.
**Table S2:** Experimental nest predation results of artificial nests at two study sites.

## Data Availability

Data used for this study are provided as the Supporting Information (Tables S1 and S2).
