# Supplementary material for: The Larger the Egg, the Safer the Nest: A Study on the Negative Correlation Between Nest Predation Rates and Egg Size of Two Tropical Phasianids in Hainan
Source: Ecol Evol. 2026 May 10;16(5):e73569. doi: 10.1002/ece3.73569 (PMC13158515; doi:10.1002/ece3.73569)
Supplement: Supplementary file 1 — Table S1: Parameters of the experimental eggs. Table S2: Experimental nest predation results of artificial nests at two study sites. [file ECE3-16-e73569-s001.zip › Table S1-S2.docx]

**TABLE S1.** Parameters of the experimental eggs

| **Serial number** | **Experimental egg** | **Egg weight**  **(g)** | **Egg length**  **(mm)** | **Egg breadth(mm)** | Egg volume(cm3) |
| --- | --- | --- | --- | --- | --- |
| 1 | Quail egg | 11.32 | 32.02 | 25.54 | 10.65 |
| 2 | Quail egg | 11.99 | 32.52 | 26.16 | 11.35 |
| 3 | Quail egg | 11.48 | 32.05 | 25.96 | 11.02 |
| 4 | Quail egg | 11.58 | 32.17 | 25.49 | 10.66 |
| 5 | Quail egg | 9.13 | 29.50 | 23.73 | 8.47 |
| 6 | Quail egg | 10.92 | 30.67 | 25.10 | 9.85 |
| 7 | Quail egg | 11.10 | 32.96 | 24.97 | 10.48 |
| 8 | Quail egg | 12.19 | 33.34 | 25.66 | 11.20 |
| 9 | Quail egg | 11.30 | 32.81 | 25.18 | 10.61 |
| 10 | Quail egg | 12.14 | 33.09 | 26.02 | 11.43 |
| 11 | Quail egg | 10.80 | 30.94 | 24.81 | 9.71 |
| 12 | Quail egg | 11.12 | 32.78 | 24.78 | 10.27 |
| 13 | Quail egg | 10.25 | 30.24 | 24.70 | 9.41 |
| 14 | Quail egg | 11.73 | 33.16 | 24.76 | 10.37 |
| 15 | Quail egg | 10.76 | 33.59 | 23.98 | 9.85 |
| 16 | Quail egg | 10.41 | 30.69 | 24.48 | 9.38 |
| 17 | Quail egg | 10.95 | 31.89 | 24.72 | 9.94 |
| 18 | Quail egg | 9.61 | 30.49 | 23.92 | 8.90 |
| 19 | Quail egg | 11.95 | 32.87 | 25.69 | 11.06 |
| 20 | Quail egg | 8.26 | 28.15 | 22.83 | 7.48 |
| 21 | Chinese Francolin egg | 17.12 | 38.40 | 28.19 | 15.56 |
| 22 | Chinese Francolin egg | 18.89 | 38.76 | 29.53 | 17.24 |
| 23 | Chinese Francolin egg | 19.28 | 40.95 | 29.12 | 17.71 |
| 24 | Chinese Francolin egg | 20.13 | 39.72 | 30.54 | 18.89 |
| 25 | Chinese Francolin egg | 17.25 | 39.17 | 28.45 | 16.17 |
| 26 | Chinese Francolin egg | 18.00 | 38.29 | 29.07 | 16.50 |
| 27 | Chinese Francolin egg | 22.47 | 39.77 | 31.73 | 20.42 |
| 28 | Chinese Francolin egg | 17.65 | 36.79 | 29.79 | 16.65 |
| 29 | Chinese Francolin egg | 17.59 | 38.09 | 28.98 | 16.31 |
| 30 | Chinese Francolin egg | 15.07 | 36.80 | 27.08 | 13.76 |
| 31 | Chinese Francolin egg | 19.57 | 39.94 | 30.01 | 18.34 |
| 32 | Chinese Francolin egg | 20.31 | 39.97 | 30.12 | 18.49 |
| 33 | Chinese Francolin egg | 18.64 | 39.92 | 29.06 | 17.19 |
| 34 | Chinese Francolin egg | 17.90 | 37.88 | 29.10 | 16.36 |
| 35 | Chinese Francolin egg | 18.49 | 39.37 | 29.17 | 17.08 |
| 36 | Chinese Francolin egg | 17.29 | 37.43 | 28.94 | 15.99 |
| 37 | Chinese Francolin egg | 18.07 | 37.20 | 29.43 | 16.43 |
| 38 | Chinese Francolin egg | 21.35 | 40.82 | 30.54 | 19.42 |
| 39 | Chinese Francolin egg | 17.03 | 36.45 | 29.07 | 15.71 |
| 40 | Chinese Francolin egg | 20.92 | 41.08 | 30.81 | 19.89 |
| 41 | Chicken egg | 58.53 | 55.70 | 43.56 | 53.90 |
| 42 | Chicken egg | 61.63 | 56.43 | 44.19 | 56.20 |
| 43 | Chicken egg | 63.04 | 56.88 | 44.64 | 57.81 |
| 44 | Chicken egg | 58.21 | 56.77 | 42.89 | 53.26 |
| 45 | Chicken egg | 58.21 | 54.38 | 44.16 | 54.08 |
| 46 | Chicken egg | 60.39 | 56.68 | 43.69 | 55.18 |
| 47 | Chicken egg | 60.35 | 57.85 | 43.29 | 55.29 |
| 48 | Chicken egg | 58.74 | 56.38 | 42.78 | 52.62 |
| 49 | Chicken egg | 63.18 | 55.88 | 55.39 | 87.44 |
| 50 | Chicken egg | 58.58 | 55.26 | 43.55 | 53.45 |
| 51 | Chicken egg | 53.40 | 54.30 | 42.03 | 48.92 |
| 52 | Chicken egg | 54.43 | 54.56 | 42.09 | 49.29 |
| 53 | Chicken egg | 59.32 | 59.05 | 42.25 | 53.76 |
| 54 | Chicken egg | 56.97 | 54.99 | 42.81 | 51.40 |
| 55 | Chicken egg | 62.18 | 58.43 | 43.67 | 56.83 |
| 56 | Chicken egg | 57.65 | 54.34 | 43.30 | 51.96 |
| 57 | Chicken egg | 61.48 | 57.43 | 42.33 | 52.48 |
| 58 | Chicken egg | 53.62 | 54.79 | 41.60 | 48.36 |
| 59 | Chicken egg | 58.75 | 57.66 | 42.52 | 53.17 |
| 60 | Chicken egg | 64.50 | 58.18 | 44.61 | 59.05 |
| 61 | Goose egg | 160.00 | 99.25 | 52.44 | 139.20 |
| 62 | Goose egg | 180.00 | 97.89 | 55.19 | 152.07 |
| 63 | Goose egg | 145.00 | 94.07 | 50.78 | 123.71 |
| 64 | Goose egg | 160.00 | 84.04 | 58.01 | 144.23 |
| 65 | Goose egg | 145.00 | 92.71 | 53.73 | 136.50 |
| 66 | Goose egg | 175.00 | 96.91 | 53.15 | 139.62 |
| 67 | Goose egg | 160.00 | 86.26 | 56.98 | 142.83 |
| 68 | Goose egg | 160.00 | 85.48 | 57.53 | 144.29 |
| 69 | Goose egg | 185.00 | 85.79 | 58.82 | 151.38 |
| 70 | Goose egg | 160.00 | 79.08 | 58.22 | 136.70 |
| 71 | Goose egg | 150.00 | 83.40 | 58.19 | 144.02 |
| 72 | Goose egg | 150.00 | 80.40 | 56.87 | 132.62 |
| 73 | Goose egg | 120.00 | 79.96 | 54.22 | 119.88 |
| 74 | Goose egg | 145.00 | 81.12 | 55.32 | 126.61 |
| 75 | Goose egg | 145.00 | 82.80 | 56.61 | 135.33 |
| 76 | Goose egg | 170.00 | 92.73 | 57.84 | 158.21 |
| 77 | Goose egg | 125.00 | 87.51 | 58.04 | 150.34 |
| 78 | Goose egg | 160.00 | 82.24 | 55.82 | 130.69 |
| 79 | Goose egg | 145.00 | 81.02 | 57.33 | 135.81 |
| 80 | Goose egg | 160.00 | 84.74 | 54.60 | 128.84 |

**TABLE S2.** Experimental nest predation results of artificial nests at two study sites

| **Study area** | **Group** | **Egg size grade** | **Experimental egg** | **Nest ID** | **Longitude**  **(°)** | **Latitude**  **(°)** | **Year** | **Experiment date** | **Vegetation type** | **Result** |
| --- | --- | --- | --- | --- | --- | --- | --- | --- | --- | --- |
| Datian | A | 1 | Quail egg | 001 | 108.7879094 | 19.10439055 | 2023 | 3.29 | Low-elevation tropical grasslands | Succeeded |
| Datian | A | 1 | Quail egg | 002 | 108.7890346 | 19.10520411 | 2023 | 3.29 | Artificial grasslands | Failed |
| Datian | A | 1 | Quail egg | 003 | 108.7897212 | 19.10963177 | 2023 | 3.29 | Artificial grasslands | Failed |
| Datian | A | 1 | Quail egg | 004 | 108.7814748 | 19.10482013 | 2023 | 3.29 | Plantation forests | Failed |
| Datian | A | 1 | Quail egg | 005 | 108.7780844 | 19.10011228 | 2023 | 3.31 | Low-elevation tropical grasslands | Failed |
| Datian | A | 1 | Quail egg | 006 | 108.7766468 | 19.10185097 | 2023 | 3.31 | Low-elevation tropical grasslands | Succeeded |
| Datian | A | 1 | Quail egg | 007 | 108.7795972 | 19.10030491 | 2023 | 3.31 | Plantation forests | Succeeded |
| Datian | A | 1 | Quail egg | 008 | 108.7811448 | 19.09862323 | 2023 | 3.31 | Low-elevation tropical grasslands | Failed |
| Datian | A | 1 | Quail egg | 009 | 108.7799513 | 19.10218553 | 2023 | 3.31 | Low-elevation tropical grasslands | Failed |
| Datian | A | 1 | Quail egg | 010 | 108.7799513 | 19.10218553 | 2023 | 3.31 | Low-elevation tropical grasslands | Failed |
| Datian | A | 1 | Quail egg | 011 | 108.7924544 | 19.11451549 | 2023 | 3.31 | Low-elevation tropical grasslands | Failed |
| Datian | A | 1 | Quail egg | 012 | 108.7943078 | 19.11584601 | 2023 | 3.31 | Artificial grasslands | Failed |
| Datian | A | 1 | Quail egg | 013 | 108.7926046 | 19.1082087 | 2023 | 4.01 | Low-elevation tropical grasslands | Failed |
| Datian | A | 1 | Quail egg | 014 | 108.797454 | 19.11430008 | 2023 | 4.01 | Low-elevation tropical grasslands | Failed |
| Datian | A | 1 | Quail egg | 015 | 108.8016879 | 19.1153797 | 2023 | 4.02 | Low-elevation tropical grasslands | Failed |
| Datian | A | 1 | Quail egg | 016 | 108.8042306 | 19.11513133 | 2023 | 4.02 | Low-elevation tropical grasslands | Failed |
| Datian | A | 1 | Quail egg | 017 | 108.803658 | 19.11401877 | 2023 | 4.02 | Low-elevation tropical grasslands | Failed |
| Datian | A | 1 | Quail egg | 018 | 108.80887 | 19.11337378 | 2023 | 4.02 | Low-elevation tropical grasslands | Failed |
| Datian | A | 1 | Quail egg | 019 | 108.784334 | 19.10243771 | 2023 | 4.03 | Low-elevation tropical grasslands | Failed |
| Datian | A | 1 | Quail egg | 020 | 108.7843769 | 19.09424849 | 2023 | 4.03 | Low-elevation tropical grasslands | Failed |
| Datian | A | 1 | Quail egg | 021 | 108.8059151 | 19.11155496 | 2023 | 4.04 | Low-elevation tropical grasslands | Failed |
| Datian | B | 2 | Chinese Francolin egg | 001 | 108.7934533 | 19.11500316 | 2023 | 3.10 | Low-elevation tropical grasslands | Succeeded |
| Datian | B | 2 | Chinese Francolin egg | 002 | 108.7953255 | 19.11544485 | 2023 | 3.10 | Low-elevation tropical grasslands | Failed |
| Datian | B | 2 | Chinese Francolin egg | 003 | 108.8000561 | 19.11753887 | 2023 | 3.10 | Low-elevation tropical grasslands | Failed |
| Datian | B | 2 | Chinese Francolin egg | 004 | 108.8019923 | 19.11715051 | 2023 | 3.10 | Low-elevation tropical grasslands | Failed |
| Datian | B | 2 | Chinese Francolin egg | 005 | 108.8044731 | 19.1172755 | 2023 | 3.10 | Low-elevation tropical grasslands | Succeeded |
| Datian | B | 2 | Chinese Francolin egg | 006 | 108.8027121 | 19.11884803 | 2023 | 3.10 | Low-elevation tropical grasslands | Succeeded |
| Datian | B | 2 | Chinese Francolin egg | 007 | 108.8032944 | 19.11330345 | 2023 | 3.10 | Low-elevation tropical grasslands | Failed |
| Datian | B | 2 | Chinese Francolin egg | 008 | 108.8041358 | 19.11155793 | 2023 | 3.10 | Low-elevation tropical grasslands | Failed |
| Datian | B | 2 | Chinese Francolin egg | 009 | 108.8058195 | 19.11325333 | 2023 | 3.10 | Low-elevation tropical grasslands | Failed |
| Datian | B | 2 | Chinese Francolin egg | 010 | 108.8058998 | 19.11154436 | 2023 | 3.10 | Low-elevation tropical grasslands | Failed |
| Datian | B | 2 | Chinese Francolin egg | 011 | 108.8089019 | 19.11956341 | 2023 | 3.11 | Low-elevation tropical grasslands | Failed |
| Datian | B | 2 | Chinese Francolin egg | 012 | 108.8056869 | 19.11727575 | 2023 | 3.11 | Low-elevation tropical grasslands | Failed |
| Datian | B | 2 | Chinese Francolin egg | 013 | 108.8060489 | 19.11519739 | 2023 | 3.11 | Low-elevation tropical grasslands | Failed |
| Datian | B | 2 | Chinese Francolin egg | 014 | 108.8100025 | 19.11744919 | 2023 | 3.11 | Low-elevation tropical grasslands | Succeeded |
| Datian | B | 2 | Chinese Francolin egg | 015 | 108.7916469 | 19.10922507 | 2023 | 3.11 | Artificial grasslands | Failed |
| Datian | B | 2 | Chinese Francolin egg | 016 | 108.7933848 | 19.10830387 | 2023 | 3.11 | Plantation forests | Succeeded |
| Datian | B | 2 | Chinese Francolin egg | 017 | 108.7911631 | 19.10646384 | 2023 | 3.11 | Artificial grasslands | Failed |
| Datian | B | 2 | Chinese Francolin egg | 018 | 108.7842195 | 19.10866232 | 2023 | 3.11 | Low-elevation tropical grasslands | Failed |
| Datian | B | 2 | Chinese Francolin egg | 019 | 108.7868831 | 19.10652226 | 2023 | 3.10 | Artificial grasslands | Failed |
| Datian | B | 2 | Chinese Francolin egg | 020 | 108.785877 | 19.10851357 | 2023 | 3.11 | Artificial grasslands | Succeeded |
| Datian | B | 2 | Chinese Francolin egg | 021 | 108.8068848 | 19.11931051 | 2023 | 3.11 | Low-elevation tropical grasslands | Succeeded |
| Datian | B | 2 | Chinese Francolin egg | 022 | 108.7845021 | 19.10262762 | 2023 | 3.14 | Low-elevation tropical grasslands | Succeeded |
| Datian | B | 2 | Chinese Francolin egg | 023 | 108.7788915 | 19.09751296 | 2023 | 3.14 | Low-elevation tropical grasslands | Failed |
| Datian | B | 2 | Chinese Francolin egg | 024 | 108.7805613 | 19.09563376 | 2023 | 3.14 | Low-elevation tropical grasslands | Failed |
| Datian | B | 2 | Chinese Francolin egg | 025 | 108.7829247 | 19.09742609 | 2023 | 3.14 | Plantation forests | Failed |
| Datian | B | 2 | Chinese Francolin egg | 026 | 108.7812649 | 19.09870778 | 2023 | 3.14 | Low-elevation tropical grasslands | Failed |
| Datian | B | 2 | Chinese Francolin egg | 027 | 108.7810742 | 19.10536312 | 2023 | 3.14 | Low-elevation tropical grasslands | Failed |
| Datian | B | 2 | Chinese Francolin egg | 028 | 108.7848212 | 19.10778454 | 2023 | 3.14 | Low-elevation tropical grasslands | Failed |
| Datian | C | 3 | Chicken egg | 001 | 108.782203 | 19.10809971 | 2023 | 3.29 | Plantation forests | Failed |
| Datian | C | 3 | Chicken egg | 002 | 108.7811797 | 19.10684137 | 2023 | 3.29 | Low-elevation tropical grasslands | Failed |
| Datian | C | 3 | Chicken egg | 003 | 108.7864342 | 19.10069902 | 2023 | 3.29 | Shrublands | Succeeded |
| Datian | C | 3 | Chicken egg | 004 | 108.7873166 | 19.10850649 | 2023 | 3.29 | Artificial grasslands | Failed |
| Datian | C | 3 | Chicken egg | 005 | 108.7894382 | 19.11188738 | 2023 | 3.29 | Artificial grasslands | Failed |
| Datian | C | 3 | Chicken egg | 006 | 108.7795328 | 19.10454768 | 2023 | 3.31 | Plantation forests | Failed |
| Datian | C | 3 | Chicken egg | 007 | 108.7781005 | 19.10305106 | 2023 | 3.31 | Plantation forests | Succeeded |
| Datian | C | 3 | Chicken egg | 008 | 108.77778 | 19.10635855 | 2023 | 3.31 | Plantation forests | Failed |
| Datian | C | 3 | Chicken egg | 009 | 108.7820648 | 19.10054949 | 2023 | 3.31 | Low-elevation tropical grasslands | Failed |
| Datian | C | 3 | Chicken egg | 010 | 108.7971335 | 19.11829033 | 2023 | 3.31 | Low-elevation tropical grasslands | Succeeded |
| Datian | C | 3 | Chicken egg | 011 | 108.7948818 | 19.11446354 | 2023 | 3.31 | Low-elevation tropical grasslands | Succeeded |
| Datian | C | 3 | Chicken egg | 012 | 108.797108 | 19.11086728 | 2023 | 3.31 | Shrublands | Failed |
| Datian | C | 3 | Chicken egg | 013 | 108.7927199 | 19.11077732 | 2023 | 4.01 | Shrublands | Failed |
| Datian | C | 3 | Chicken egg | 014 | 108.7996682 | 19.11762508 | 2023 | 4.01 | Low-elevation tropical grasslands | Succeeded |
| Datian | C | 3 | Chicken egg | 015 | 108.8041582 | 19.11220163 | 2023 | 4.02 | Low-elevation tropical grasslands | Failed |
| Datian | C | 3 | Chicken egg | 016 | 108.8063697 | 19.11125251 | 2023 | 4.02 | Low-elevation tropical grasslands | Failed |
| Datian | C | 3 | Chicken egg | 017 | 108.8066781 | 19.11555203 | 2023 | 4.02 | Low-elevation tropical grasslands | Failed |
| Datian | C | 3 | Chicken egg | 018 | 108.7830988 | 19.1041409 | 2023 | 4.03 | Low-elevation tropical grasslands | Failed |
| Datian | C | 3 | Chicken egg | 019 | 108.7821064 | 19.09632816 | 2023 | 4.03 | Low-elevation tropical grasslands | Succeeded |
| Datian | C | 3 | Chicken egg | 020 | 108.8045914 | 19.11762002 | 2023 | 4.04 | Low-elevation tropical grasslands | Failed |
| Datian | D | 4 | Goose egg | 001 | 108.7831645 | 19.10666016 | 2023 | 3.29 | Low-elevation tropical grasslands | Failed |
| Datian | D | 4 | Goose egg | 002 | 108.7865307 | 19.10321834 | 2023 | 3.29 | Shrublands | Succeeded |
| Datian | D | 4 | Goose egg | 003 | 108.7861753 | 19.10751046 | 2023 | 3.29 | Artificial grasslands | Failed |
| Datian | D | 4 | Goose egg | 004 | 108.7872603 | 19.11137543 | 2023 | 3.29 | Artificial grasslands | Failed |
| Datian | D | 4 | Goose egg | 005 | 108.7904038 | 19.10783993 | 2023 | 3.29 | Plantation forests | Failed |
| Datian | D | 4 | Goose egg | 006 | 108.7808511 | 19.10328424 | 2023 | 3.31 | Low-elevation tropical grasslands | Succeeded |
| Datian | D | 4 | Goose egg | 007 | 108.7761761 | 19.10492025 | 2023 | 3.31 | Plantation forests | Succeeded |
| Datian | D | 4 | Goose egg | 008 | 108.7794725 | 19.09812391 | 2023 | 3.31 | Plantation forests | Succeeded |
| Datian | D | 4 | Goose egg | 009 | 108.7908853 | 19.11358919 | 2023 | 3.31 | Low-elevation tropical grasslands | Failed |
| Datian | D | 4 | Goose egg | 010 | 108.7946739 | 19.11286057 | 2023 | 3.31 | Shrublands | Succeeded |
| Datian | D | 4 | Goose egg | 011 | 108.7911307 | 19.10634588 | 2023 | 3.31 | Artificial grasslands | Succeeded |
| Datian | D | 4 | Goose egg | 012 | 108.7946806 | 19.11086855 | 2023 | 4.01 | Shrublands | Succeeded |
| Datian | D | 4 | Goose egg | 013 | 108.7978201 | 19.1166836 | 2023 | 4.01 | Low-elevation tropical grasslands | Failed |
| Datian | D | 4 | Goose egg | 014 | 108.7997661 | 19.11612605 | 2023 | 4.02 | Low-elevation tropical grasslands | Failed |
| Datian | D | 4 | Goose egg | 015 | 108.8053209 | 19.10971033 | 2023 | 4.02 | Low-elevation tropical grasslands | Failed |
| Datian | D | 4 | Goose egg | 016 | 108.8064743 | 19.11335604 | 2023 | 4.02 | Low-elevation tropical grasslands | Failed |
| Datian | D | 4 | Goose egg | 017 | 108.8084538 | 19.11538857 | 2023 | 4.02 | Low-elevation tropical grasslands | Failed |
| Datian | D | 4 | Goose egg | 018 | 108.7827957 | 19.09827472 | 2023 | 4.03 | Low-elevation tropical grasslands | Succeeded |
| Datian | D | 4 | Goose egg | 019 | 108.8018622 | 19.11789499 | 2023 | 4.04 | Low-elevation tropical grasslands | Succeeded |
| Datian | D | 4 | Goose egg | 020 | 108.8066643 | 19.11700956 | 2023 | 4.04 | Low-elevation tropical grasslands | Succeeded |
| Bangxi | A | 1 | Quail egg | 001 | 109.1074773 | 19.39141073 | 2023 | 6.01 | Seasonal evergreen rainforests | Failed |
| Bangxi | A | 1 | Quail egg | 002 | 109.1066442 | 19.39595108 | 2023 | 6.01 | Seasonal evergreen rainforests | Failed |
| Bangxi | A | 1 | Quail egg | 003 | 109.1019104 | 19.39272250 | 2023 | 6.01 | Seasonal evergreen rainforests | Failed |
| Bangxi | A | 1 | Quail egg | 004 | 109.1027922 | 19.39019306 | 2023 | 6.01 | Seasonal evergreen rainforests | Failed |
| Bangxi | A | 1 | Quail egg | 005 | 109.0998987 | 19.39390353 | 2023 | 6.01 | Seasonal evergreen rainforests | Failed |
| Bangxi | A | 1 | Quail egg | 006 | 109.1022461 | 19.39386862 | 2023 | 6.01 | Seasonal evergreen rainforests | Failed |
| Bangxi | A | 1 | Quail egg | 007 | 109.1020946 | 19.39846378 | 2023 | 6.01 | Seasonal deciduous rainforests | Failed |
| Bangxi | A | 1 | Quail egg | 008 | 109.106105 | 19.39836294 | 2023 | 6.01 | Seasonal deciduous rainforests | Failed |
| Bangxi | A | 1 | Quail egg | 009 | 109.1103693 | 19.39520315 | 2023 | 6.01 | Seasonal deciduous rainforests | Failed |
| Bangxi | A | 1 | Quail egg | 010 | 109.1103669 | 19.39396291 | 2023 | 6.01 | Seasonal deciduous rainforests | Succeeded |
| Bangxi | A | 1 | Quail egg | 011 | 109.1106408 | 19.39189619 | 2023 | 6.01 | Seasonal deciduous rainforests | Failed |
| Bangxi | A | 1 | Quail egg | 012 | 109.1052254 | 19.38867477 | 2023 | 8.17 | Seasonal evergreen rainforests | Failed |
| Bangxi | A | 1 | Quail egg | 013 | 109.1072049 | 19.39472538 | 2023 | 8.17 | Seasonal evergreen rainforests | Succeeded |
| Bangxi | A | 1 | Quail egg | 014 | 109.1013993 | 19.39084937 | 2023 | 8.17 | Seasonal evergreen rainforests | Failed |
| Bangxi | A | 1 | Quail egg | 015 | 109.1033506 | 19.39426998 | 2023 | 8.17 | Seasonal evergreen rainforests | Failed |
| Bangxi | A | 1 | Quail egg | 016 | 109.1014462 | 19.3979726 | 2023 | 8.17 | Tropical grasslands with sparse trees | Failed |
| Bangxi | A | 1 | Quail egg | 017 | 109.1054293 | 19.39858232 | 2023 | 8.17 | Seasonal deciduous rainforests | Failed |
| Bangxi | A | 1 | Quail egg | 018 | 109.1104651 | 19.39501633 | 2023 | 8.17 | Seasonal deciduous rainforests | Failed |
| Bangxi | A | 1 | Quail egg | 019 | 109.1092192 | 19.39287467 | 2023 | 8.17 | Tropical grasslands with sparse trees | Failed |
| Bangxi | A | 1 | Quail egg | 020 | 109.1052563 | 19.39738439 | 2023 | 8.17 | Seasonal deciduous rainforests | Succeeded |
| Bangxi | A | 1 | Quail egg | 021 | 109.1024748 | 19.39362862 | 2023 | 8.17 | Tropical grasslands with sparse trees | Succeeded |
| Bangxi | B | 2 | Chinese Francolin egg | 001 | 109.1049719 | 19.390141 | 2023 | 5.23 | Tropical grasslands with sparse trees | Failed |
| Bangxi | B | 2 | Chinese Francolin egg | 002 | 109.1069549 | 19.39067912 | 2023 | 5.23 | Tropical grasslands with sparse trees | Succeeded |
| Bangxi | B | 2 | Chinese Francolin egg | 003 | 109.1058183 | 19.39135344 | 2023 | 5.23 | Tropical grasslands with sparse trees | Failed |
| Bangxi | B | 2 | Chinese Francolin egg | 004 | 109.1067262 | 19.3923718 | 2023 | 5.23 | Tropical grasslands with sparse trees | Succeeded |
| Bangxi | B | 2 | Chinese Francolin egg | 005 | 109.1061941 | 19.39372444 | 2023 | 5.23 | Tropical grasslands with sparse trees | Failed |
| Bangxi | B | 2 | Chinese Francolin egg | 006 | 109.1064501 | 19.39466401 | 2023 | 5.23 | Tropical grasslands with sparse trees | Failed |
| Bangxi | B | 2 | Chinese Francolin egg | 007 | 109.1067083 | 19.39577091 | 2023 | 5.23 | Tropical grasslands with sparse trees | Failed |
| Bangxi | B | 2 | Chinese Francolin egg | 008 | 109.104675 | 19.39504511 | 2023 | 5.23 | Tropical grasslands with sparse trees | Succeeded |
| Bangxi | B | 2 | Chinese Francolin egg | 009 | 109.1024033 | 19.39172164 | 2023 | 5.23 | Tropical grasslands with sparse trees | Succeeded |
| Bangxi | B | 2 | Chinese Francolin egg | 010 | 109.1039235 | 19.39022657 | 2023 | 5.23 | Tropical grasslands with sparse trees | Succeeded |
| Bangxi | B | 2 | Chinese Francolin egg | 011 | 109.1046319 | 19.38822857 | 2023 | 5.23 | Tropical grasslands with sparse trees | Failed |
| Bangxi | B | 2 | Chinese Francolin egg | 012 | 109.1059085 | 19.38771073 | 2023 | 5.23 | Tropical grasslands with sparse trees | Succeeded |
| Bangxi | B | 2 | Chinese Francolin egg | 013 | 109.1012079 | 19.39194553 | 2023 | 5.23 | Tropical grasslands with sparse trees | Succeeded |
| Bangxi | B | 2 | Chinese Francolin egg | 014 | 109.1019025 | 19.39440352 | 2023 | 5.23 | Tropical grasslands with sparse trees | Failed |
| Bangxi | B | 2 | Chinese Francolin egg | 015 | 109.1020168 | 19.39294992 | 2023 | 5.23 | Tropical grasslands with sparse trees | Failed |
| Bangxi | C | 3 | Chicken egg | 001 | 109.10561794 | 19.39008275 | 2023 | 6.01 | Seasonal evergreen rainforests | Succeeded |
| Bangxi | C | 3 | Chicken egg | 002 | 109.11051140 | 19.39263308 | 2023 | 6.01 | Seasonal deciduous rainforests | Succeeded |
| Bangxi | C | 3 | Chicken egg | 003 | 109.10304980 | 19.39438318 | 2023 | 6.01 | Tropical grasslands with sparse trees | Succeeded |
| Bangxi | C | 3 | Chicken egg | 004 | 109.10432655 | 19.38858462 | 2023 | 6.01 | Seasonal evergreen rainforests | Failed |
| Bangxi | C | 3 | Chicken egg | 005 | 109.09951151 | 19.39219718 | 2023 | 6.01 | Seasonal evergreen rainforests | Failed |
| Bangxi | C | 3 | Chicken egg | 006 | 109.10073382 | 19.39232452 | 2023 | 6.01 | Seasonal evergreen rainforests | Failed |
| Bangxi | C | 3 | Chicken egg | 007 | 109.10231835 | 19.39750628 | 2023 | 6.01 | Tropical grasslands with sparse trees | Succeeded |
| Bangxi | C | 3 | Chicken egg | 008 | 109.10462602 | 19.39813519 | 2023 | 6.01 | Seasonal deciduous rainforests | Failed |
| Bangxi | C | 3 | Chicken egg | 009 | 109.10873385 | 19.39603889 | 2023 | 6.01 | Seasonal deciduous rainforests | Failed |
| Bangxi | C | 3 | Chicken egg | 010 | 109.11125350 | 19.39630202 | 2023 | 6.01 | Seasonal deciduous rainforests | Failed |
| Bangxi | C | 3 | Chicken egg | 011 | 109.11092641 | 19.39347678 | 2023 | 6.01 | Seasonal deciduous rainforests | Failed |
| Bangxi | C | 3 | Chicken egg | 012 | 109.1032258 | 19.38954132 | 2023 | 8.17 | Tropical grasslands with sparse trees | Succeeded |
| Bangxi | C | 3 | Chicken egg | 013 | 109.1062299 | 19.39234842 | 2023 | 8.17 | Seasonal evergreen rainforests | Succeeded |
| Bangxi | C | 3 | Chicken egg | 014 | 109.1004766 | 19.39259257 | 2023 | 8.17 | Seasonal evergreen rainforests | Succeeded |
| Bangxi | C | 3 | Chicken egg | 015 | 109.1028517 | 19.39209415 | 2023 | 8.17 | Seasonal evergreen rainforests | Succeeded |
| Bangxi | C | 3 | Chicken egg | 016 | 109.1022723 | 19.39616621 | 2023 | 8.17 | Seasonal deciduous rainforests | Succeeded |
| Bangxi | C | 3 | Chicken egg | 017 | 109.1084857 | 19.39625476 | 2023 | 8.17 | Seasonal deciduous rainforests | Succeeded |
| Bangxi | C | 3 | Chicken egg | 018 | 109.1109493 | 19.39360205 | 2023 | 8.17 | Seasonal deciduous rainforests | Succeeded |
| Bangxi | C | 3 | Chicken egg | 019 | 109.1075952 | 19.39676202 | 2023 | 8.17 | Seasonal deciduous rainforests | Succeeded |
| Bangxi | C | 3 | Chicken egg | 020 | 109.1040815 | 19.39602706 | 2023 | 8.17 | Seasonal deciduous rainforests | Succeeded |
| Bangxi | C | 3 | Chicken egg | 021 | 109.1024279 | 19.39741475 | 2023 | 8.17 | Tropical grasslands with sparse trees | Succeeded |
| Bangxi | D | 4 | Goose egg | 001 | 109.10449756 | 19.39103563 | 2023 | 6.01 | Seasonal evergreen rainforests | Succeeded |
| Bangxi | D | 4 | Goose egg | 002 | 109.10518037 | 19.39242983 | 2023 | 6.01 | Seasonal evergreen rainforests | Succeeded |
| Bangxi | D | 4 | Goose egg | 003 | 109.10463109 | 19.39551071 | 2023 | 6.01 | Seasonal evergreen rainforests | Succeeded |
| Bangxi | D | 4 | Goose egg | 004 | 109.10583172 | 19.38821419 | 2023 | 6.01 | Seasonal evergreen rainforests | Succeeded |
| Bangxi | D | 4 | Goose egg | 005 | 109.10106536 | 19.39118982 | 2023 | 6.01 | Seasonal evergreen rainforests | Succeeded |
| Bangxi | D | 4 | Goose egg | 006 | 109.10105917 | 19.39381341 | 2023 | 6.01 | Seasonal evergreen rainforests | Succeeded |
| Bangxi | D | 4 | Goose egg | 007 | 109.1022382 | 19.39623622 | 2023 | 6.01 | Seasonal deciduous rainforests | Succeeded |
| Bangxi | D | 4 | Goose egg | 008 | 109.10337309 | 19.39737787 | 2023 | 6.01 | Seasonal deciduous rainforests | Succeeded |
| Bangxi | D | 4 | Goose egg | 009 | 109.10766805 | 19.39736267 | 2023 | 6.01 | Seasonal deciduous rainforests | Failed |
| Bangxi | D | 4 | Goose egg | 010 | 109.11218557 | 19.39503692 | 2023 | 6.01 | Seasonal deciduous rainforests | Succeeded |
| Bangxi | D | 4 | Goose egg | 011 | 109.10987515 | 19.3928642 | 2023 | 6.01 | Tropical grasslands with sparse trees | Succeeded |
| Bangxi | D | 4 | Goose egg | 012 | 109.1047198 | 19.3906963 | 2023 | 8.17 | Seasonal evergreen rainforests | Succeeded |
| Bangxi | D | 4 | Goose egg | 013 | 109.1068763 | 19.39023077 | 2023 | 8.17 | Tropical grasslands with sparse trees | Succeeded |
| Bangxi | D | 4 | Goose egg | 014 | 109.1066309 | 19.39438383 | 2023 | 8.17 | Seasonal evergreen rainforests | Succeeded |
| Bangxi | D | 4 | Goose egg | 015 | 109.1012423 | 19.39448503 | 2023 | 8.17 | Seasonal evergreen rainforests | Succeeded |
| Bangxi | D | 4 | Goose egg | 016 | 109.1054937 | 19.39579177 | 2023 | 8.17 | Seasonal evergreen rainforests | Succeeded |
| Bangxi | D | 4 | Goose egg | 017 | 109.1035853 | 19.39738186 | 2023 | 8.17 | Seasonal deciduous rainforests | Succeeded |
| Bangxi | D | 4 | Goose egg | 018 | 109.1071633 | 19.3978752 | 2023 | 8.17 | Seasonal deciduous rainforests | Succeeded |
| Bangxi | D | 4 | Goose egg | 019 | 109.1091012 | 19.39447617 | 2023 | 8.17 | Seasonal deciduous rainforests | Succeeded |
| Bangxi | D | 4 | Goose egg | 020 | 109.102794 | 19.39547173 | 2023 | 8.17 | Seasonal evergreen rainforests | Succeeded |
| Bangxi | D | 4 | Goose egg | 021 | 109.1014355 | 19.3961485 | 2023 | 8.17 | Seasonal deciduous rainforests | Succeeded |
